# Identification of H-2d Restricted T Cell Epitope of Foot-and-mouth Disease Virus Structural Protein VP1

**DOI:** 10.1186/1743-422X-8-426

**Published:** 2011-09-07

**Authors:** Xin-Sheng Liu, Yong-Lu Wang, Yong-Guang Zhang, Yu-Zhen Fang, Li Pan, Jian-Liang Lu, Peng Zhou, Zhong-Wang Zhang, Shou-Tian Jiang

**Affiliations:** 1 State Key Laboratory of Veterinary Etiological Biology, National Foot and Mouth Disease Reference Laboratory, Lanzhou Veterinary Research Institute, Chinese Academy of Agricultural Sciences, Lanzhou 730046, China

## Abstract

**Background:**

Foot-and-mouth disease (FMD) is a highly contagious and devastating disease affecting livestock that causes significant financial losses. Therefore, safer and more effective vaccines are required against Foot-and-mouth disease virus(FMDV). The purpose of this study is to screen and identify an H-2d restricted T cell epitope from the virus structural protein VP1, which is present with FMD. We therefore provide a method and basis for studying a specific FMDV T cell epitope.

**Results:**

A codon-optimized expression method was adopted for effective expression of VP1 protein in colon bacillus. We used foot-and-mouth disease standard positive serum was used for Western blot detection of its immunogenicity. The VP1 protein was used for immunizing BALB/c mice, and spleen lymphocytes were isolated. Then, a common in vitro training stimulus was conducted for potential H-2Dd, H-2Kd and H-2Ld restricted T cell epitope on VP1 proteins that were predicted and synthesized by using a bioinformatics method. The H-2Kd restricted T cell epitope pK1 (AYHKGPFTRL) and the H-2Dd restricted T cell epitope pD7 (GFIMDRFVKI) were identified using lymphocyte proliferation assays and IFN-γ ELISPOT experiments.

**Conclusions:**

The results of this study lay foundation for studying the FMDV immune process, vaccine development, among other things. These results also showed that, to identify viral T cell epitopes, the combined application of bioinformatics and molecular biology methods is effective.

## Background

FMD is an acute, febrile, highly contagious zoonotic disease [1] that is caused by the FMDV and that mainly harms cloven-hoofed animals. The pathogen belongs to the genus FMDV of the small RNA virus branch. Cross-protection tests and serological tests have confirmed that FMDV has seven serotypes, namely O, A, C (European type), Asia 1 (Asia 1 type) and the STA1, STA2, STA3 (South African type) [2]. FMDV antigen has a complex structure and a wide range of antigenic variation, but it does not possess cross protection among reaction types, seven serotypes have a great amount of antigenic difference, which makes it more difficult for vaccine prevention [3,4]. Currently, inactivated/attenuated pathogens or the complete viral antigens are used as a vaccine. The former has a good effect, but has biological safety problems; while the effect of the latter is often very limited. Thus, epitope-based vaccine design has become the priority and hot spot of current FMD vaccine research. At present, there are some more successful reports about research on the FMD epitope vaccine [5-9], but all of these are based on determining B and T cell epitopes from the FMDV structural and non-structural proteins. Identification of FMDV epitopes is also a very active area of research in recent years. Previous research has identified a number of effective FMDV epitopes [10-22], which provides important keys for developing epitope vaccines with higher immunogenicity and protective capabilities.

FMDV particles are mainly composed of 4 capsid proteins (i.e., VP1, VP2, VP3 and VP4) with 60 respective copies. The structural protein VP1 contains major antigenic sites of the FMDV. The VP1 genes mutate easily, which often leads to toxic antigenic strain and virulence changes. Additionally, it is the main reason for polymorphic and multi-subtype FMDV [23]. Therefore, it is particularly important for developing research on antigen epitopes regarding the FMDV structural protein VP1.

It is generally believed that anti-infection immunity against FMDV is mainly related to high levels of antibodies, but with increased research it has become evident that other parts of the cellular immune response play an important role in protection against FMD. The production of neutralizing antibodies in FMDV infection requires B cell epitopes and T cell epitopes. The vaccine cannot elicit a protective immune response without T cell epitopes [24]. Cytotoxic T lymphocytes (CTL) are one of the immune cells needed for the body to effectively control viral infection. MHC I molecule restricted antigen-specific CTL responses are important for viral clearance, to control viral replication, and to prevent diffusion during viral infection. Previous studies on FMDV antigen epitopes have mostly concentrated on the study of B cell epitopes and assistant T cell epitopes. However there is limited research on FMDV cytotoxic T lymphocyte epitopes and its effect on protection against FMDV. So far, the only discovered cytotoxic T lymphocyte epitopes on the FMDV structural protein VP1 are two pig SLA molecule restricted cytotoxic T lymphocyte epitopes [22]. Therefore, an in-depth study on the FMDV CTL T cell epitopes has important significance to accurately map CTL epitopes in detail for FMDV. This would illuminate the importance of the cellular immune response, pathogenesis of the FMDV, and provide insight in to developing epitope vaccines.

Therefore, the purpose of this study is to identify CTL T cell epitopes on the FMDV structural protein VP1. This would provide clues and help for future studies. We combined bioinformatic predictions and experimental validation, a method which is widely used and has successfully identified many antigen epitopes, for the screening and identification of H-2 restricted T cell epitopes on the FMDV structural protein VP1 [25-29]. Additionally, it was used to successfully identify possible CTL T cell epitopes on two FMDV structural proteins VP1.

## Results

### VP1 Antigen Protein Cloning, Expression and Immunogenicity Analysis

**Figure 1:**
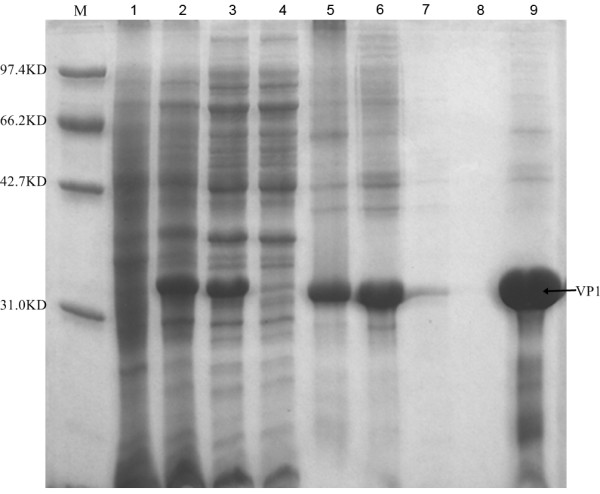
**SDS-PAGE Results of VP1 Protein**. These results show that the structural protein VP1 after optimal expression has the size of 33 kDa, which is consistent with the expected size. 1: Uninduced control; 2: 3 hour induction; 3: Disintegrating liquid with ultrasonic; 4: Clarifying after disintegrating liquid with ultrasonic and centrifugation; 5: purifying sample solution with Ni Column, dissolved inclusion bodies; 6: Purifying flowing liquid with Ni Column; 7: Purifying Ni-Denature-8.0 wash solution with Ni Column; 8: Purifying Ni-Denature-6.0 wash solution with Ni Column; 9: Purifying Ni-Denature-4.0 equate with Ni Column;

**Figure 2:**
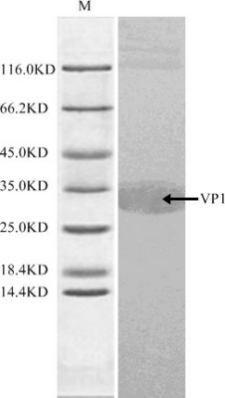
**VP1 Protein Western blot analysis; The results show that specific bands were available at a molecular weight of 33 kDa**. The expression protein and FMDV positive serum generate strong specific reactions, thereby indicating that VP1 protein with optimal expression has good immunogenicity and the antigenicity is not changed

We obtained the complete sequence of the wild-type FMDV AF/72 strain structural protein VP1 gene through cloning and sequencing. Sequence analysis results revealed that the full length VP1 was successfully cloned with all 639 nucleotides and 213 amino acids that encode the protein. The expression of an exogenous gene in E. coli was controlled by a number of factors, mainly in the transcription and translation stages, and it was also important to affect protein expression using varying passwords. Rare codons in the sequence were changed into E. coli advantage codons, thereby optimizing the codons with the wild-type VP1 gene as reference sequences. Additionally, the adaptation index of the codons was optimized, and the GC content was balanced. The synthetic optimization sequence was connected with the vector pET28a, and BL21 (DE3) host bacteria were transformed for protein expression. Concurrently, we optimized and confirmed the optimal expression conditions for the expression of recombinant proteins. The results showed that the recombinant strain optimally expressed the protein after being induced with 1 mol/L IPTG at 37°C for 3 h. After being purified by the affinity layer, VP1 protein was identified by SDS-PAGE electrophoresis, and the results showed that VP1 protein was successfully and highly expressed in E. coli with a molecular weight of approximately 33 kDa, which was consistent with the expected size (Figure 1). Western blot analysis results showed that the VP1 protein generated under conditions promoting optimal expression can generate a specific reaction with FMDV type-A standard positive sera, which further confirmed that the VP1 protein that was generated under conditions promoting optimal expression was correctly expressed in E. coli and that it has acceptable immunogenicity (Figure 2).

### CD8^+ ^T Cell Epitope Prediction Results and Selection

CTL epitope prediction refers to peptides that can be predicted by looking for the rule of combining antigen peptide with specific MHC molecules. BIMAS algorithm experimentally measured peptide-MHC complexes and established the grade affinity matrix with the dissociation half-life under pH 6.5 in a 37°C environment, thus predicting MHC restricted epitopes that have been successfully applied to the identification of a wide range of CTL epitopes [30]. This study utilizes the software to predict the H-2d restricted cytotoxic T cell epitopes of FMDV AF/72 strain structural protein VP1. A total of 30 H-2Dd, H-2Kd and H-2Ld restricted T cell epitopes were identified by the highest score and from those 10 peptides were selected as the candidates (Table 1).

**Table 1:** Amino acid sequence of the predicted VP1 protein CD8^+ ^T cell epitopes and BIMAS scores

MHC-restriction	Code	Start Position	Subsequence Residue Listing	Score
H-2Ld	pL1	103	NPTAYHKGPF	300
	pL2	117	LPYTAPHRVL	150
	pL3	89	VPNGAPETAL	150
	pL4	9	DPVTTTVENY	72
	pL5	121	APHRVLATVY	60
	pL6	110	GPFTRLALPY	60
	pL7	45	QSPTHVIDLM	37.5
	pL8	42	IPSQSPTHVI	30
	pL9	158	LPASFNFGAI	30
	pL10	188	RPLLAVKVTS	30
H-2Dd	pD1	138	TGNAGRRGDL	30
	pD2	109	KGPFTRLALP	24
	pD3	141	AGRRGDLGS	3
	pD4	62	VGALLRAATY	10
	pD5	45	QSPTHVIDLM	7.2
	pD6	41	KIPSQSPTHV	7.2
	pD7	33	GFIMDRFVKI	6
	pD8	116	ALPYTAPHRV	6
	pD9	56	THQHGLVGAL	3
	pD10	197	SQDRHKQRII	20
H-2Kd	pK1	106	AYHKGPFTRL	3456
	pK2	33	FIMDRFVKI	1920
	pK3	163	NFGAIRATVI	1152
	pK4	71	YYFSDLEIVV	720
	pK5	70	TYYFSDLEIV	600
	pK6	184	LYCPTPLLAV	600
	pK7	204	RIIAPAKQLL	115.2
	pK8	52	DLMQTHQHGL	80
	pK9	77	EIVVRHDDNL	80
	pK10	175	LLVRVKRAEL	80

### Lymphocyte *In Vitro *Proliferation Experiment for Identifying CD8^+ ^T Cell Epitopes

**Figure 3:**
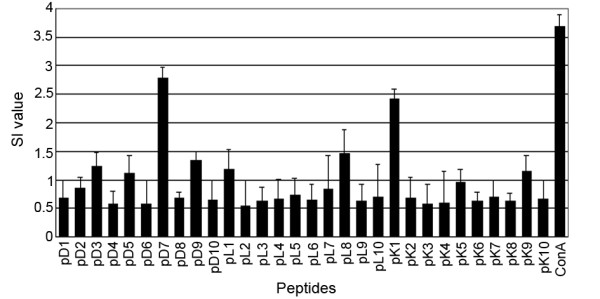
**Stimulation index (SI)**. The measured proliferative response of splenic cells from mice immunized by VP1 protein. Stimulation index is the ratio of the value between the stimulated and control cultures.

The MTS colorimetric method was used to verify CD8^+ ^T lymphocyte proliferation after mouse splenic lymphocytes were stimulated with a candidate epitope peptide section. Significant lymphocyte proliferation occurred in the positive control group, but no lymphocyte proliferation was detected in the negative control group. In the experimental group, the H-2Kd restricted T cell epitope candidate peptide pK1 and the H-2Dd restricted T cell epitope candidate peptide section pD7 stimulated the proliferation of mouse lymphocytes (SI> 2). Additionally, higher transformation capacity induced by lymphocytes could be shown, while the other candidate epitope peptide sections cannot effectively stimulate the proliferation of mouse lymphocytes sensitized by VP1 protein. In addition, these peptides were unable to stimulate the lymphocyte proliferation of PBS immunized mice (SI <2), (Figure 3). These results suggest that peptide section pK1 (AYHKGPFTRL) and pD7 (TGESADPVTT) may be epitopes of VP1's protein-specific CTL.

### INF-γ ELISPOT Experiments Further Confirmed the Results of the Proliferation Experiments

**Figure 4:**
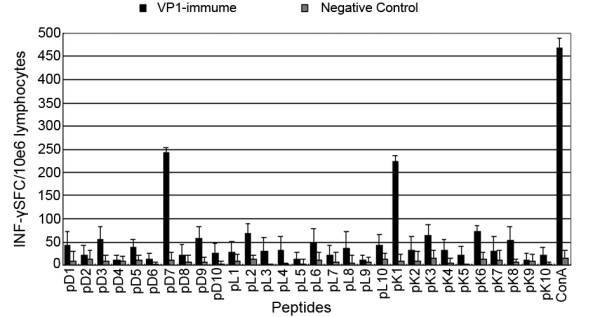
**IFN-γ ELISPOT**. The statistical results from the ELISPOT of immunized Balb/c mice splenic lymphocyte after stimulation with peptides by Analysis of Variance (ANOVA). The negative control represents lymphocytes plated with RPMI 1640 without peptides. The experimental groups are lymphocytes plated with peptides. Asterisks indicate statistical significance compared with the value of wells without peptides (P<0.01)

**Figure 5:**
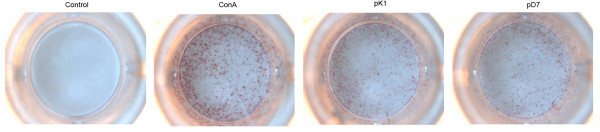
**The ELISPOT results of immunized Balb/c splenic lymphocytes after stimulation with peptides, ConA and two positive peptides (pk1: H-2K^d ^restricted, pD7: H-2D^d ^restricted)**.

IFN-γ secretion by cells was checked using ELISPOT, which further validated the results of the T cell proliferation assays. The test results show that mouse spleen lymphocyte holes stimulated by H-2Kd restricted T cell epitope pK1 (AYHKGPFTRL) and H-2Dd restricted T cell epitope pD7 (TGESADPVTT) can be seen as lymphocyte colonies within increasing IFN-γ secretion. Mouse splenic lymphocytes stimulated by other peptide sections and non-stimulated mouse splenic lymphocytes were used for detecting individual lymphocyte colony IFN-γ secretion or for detecting lymphocyte colonies that did not secrete IFN-γ (Figures 4 and 5). Anova showed that there were significant differences (P<0.01) between the spot count from mouse splenic lymphocytes that were stimulated by peptide sections pK1 and pD7 compared with lymphocytes stimulated with other peptide sections. These results confirmed the observations in the T cell proliferation experiments and further confirmed that peptide sections pK1 and pD7 may be VP1 protein-specific CTL epitopes.

## Discussion

Viral antigens are cleaved into oligopeptides of small molecules, which bind MHC-I molecules forming "antigen-MHC-I complexes". These complexes present viral proteins on the surface of antigen-presenting cells allowing CD8^+ ^T cells to specifically recognize the viral protein through their expression of the viral specific TCR. These viral peptides that are presented on MHC I to CD8^+ ^T cells represent CTL epitopes that can initiate an anti-viral response [30]. Identification of CTL epitopes has been a focus of immunology because it is the key for the research and development of immunodiagnostics, immunotherapy and new vaccines. Additionally, it is helpful for exploring and clarifying the pathogenesis of microorganisms and the subsequent immune response [31-33]. CTL play a critical role in the anti-viral response to infection in the body. However, previous studies investigating the immune response against FMD have provided little understanding of the protective role of cell cytotoxic lymphocytes against the FMDV. The research on epitopes of the FMDV has mainly focused on the study of B cell epitopes, which are associated with humoral immunity, as well as Th cell epitopes that promote the generation of high-level protective antibodies. These studies have overlooked the importance of CTL epitopes as shown with only one study on CTL epitopes of FMDV [22]. The only research report of FMDV CTL epitopes predicts possible CTL epitopes, but does not have *in vitro *tests to accurately verify these CTL epitopes as immunogenic. Therefore, our work has provided a comprehensive and detailed description of FMDV epitope mapping and has increased our understanding of the protective effects of cytotoxic lymphocytes against FMDV.

We identified candidate epitopes using bioinformatics, and candidate epitopes were scored using an epitope prediction software. Potential epitopes were determined based on their scores and the secretion of cytokines induced by the potential epitopes. As opposed to the commonly used T cell epitope identification methods, such as synthetic overlapped peptide screening method, bioinformatics technology was used to predict and analyze advantageous antigenic epitopes of the targeted protein. The epitopes can be pre-determined, thereby reducing blindness of study. Additionally, the epitopes can be validated using molecular biology, which increases the likelihood that the predicted epitopes are authentic. However, the results of the test suggest that the software prediction accuracy is low and does not completely and truly reflect actual epitopes. Therefore, it should only be used as a reference.

Many viruses have a significantly different function regarding the frequency of their codon usage compared to their host cell's codons. Viral genes may alter the expression of targeted proteins due to different codon biases when being expressed in host cells [34-36]. Therefore, we adopted a coded optimization expression method for the expression of VP1 protein in E. coli and utilized this method for preparing the FMDV structural protein VP1 from the mouse immune antigen to prevent the possibility that the FMDV structural protein VP1 alters the expression of the VP1 protein due to different codon biases in the E. coli cell expression process. Otherwise, this could result in changing identified T-cell epitopes and could limit the accuracy and efficiency of the expression of VP1. Western blot analysis showed that the VP1 protein produced under conditions that promote optimal expression can generate specific reactions with FMDV type-A standard positive sera. Additionally, we proved that VP1 protein produced under conditions that promote optimal expression was correctly expressed in E. coli and maintained immunogenicity, ensuring that the T cell epitope amino acid sequences identified in the following experiments were correct and effective.

VP1 protein with incomplete Freund's adjuvant with the same dose was used to boost the immune response 2 weeks after the primary immunization. Following the initial boost, we strengthened the immunity once every two weeks. The immunization method with a total of 3 times of strengthening immunity ensures that mice after immunization are adequate for inducing and generating enough long-term memory T cells. This was necessary to ensure that there were a sufficient number of T lymphocytes available for in vitro stimulation and to ensure more accurate test results.

Epitope selection is based on a number of factors, such as the composition of T cells and the affinity between the epitope and MHC molecules [37,38]. The frequency of CD4^+ ^T cells that secrete IFN-γ is increased with the increase in mononuclear cells. This is mediated by monocyte-derived cytokine-production [39,40]. Epitopes can be presented to CD8^+ ^T cells through monocytes. Additionally, CD8^+ ^T cells also secrete IFN-γ upon activation. In this study, the composition of the splenic lymphocytes for each mouse was the same with the constant amount, thus eliminating errors that may be caused by CD4^+ ^T cell secretion of IFN-γ. In this study, small peptides composed of ten amino acids identified through prediction synthesis were used to carry out *in vitro *stimulations of splenic lymphocytes from immunized mice. The ability of candidate epitope peptide sections to stimulate and activate mouse T lymphocytes were evaluated for cell quantity changes and cell factor secretion, respectively through T cell proliferation assays and IFN-γ Elispot experiments. The results showed that the H-2Kd restricted T cell candidate epitope peptide section pK1 (AYHKGPFTRL) and the H-2Dd restricted T cell candidate peptide epitope pD7 (TGESADPVTT) can effectively induce activated chicken T lymphocytes to produce a T cellular response. Therefore these two candidate peptide epitopes may be the mouse H-2d restricted cytotoxic T cell epitopes. The experimental results also indicate that although the two epitopes have different restrictions, they may produce T cell responses of the same type. However, this test currently only validated epitope prediction and the ability of T lymphocytes to react to the epitope. However, further tests are required to verify whether these epitopes are immunogenic. Two CTLs that were identified by these experiments could not be used because they were not the natural hosts of FMDV, such as pigs, cattle, sheep and other cloven-hoofed animals. But the process and results of these experiments provide initial evidence for studying the possible development of an epitope vaccine against FMDV. Additionally, this study is beneficial in demonstrating the effects of cytotoxic lymphocytes in protecting against FMD in their response against the FMDV, which is possible through the identification of CTL epitopes in mice, and this is a normal model for the study of FMDV.

FMDV strain AF/72 that was used in these experiments is a vaccine strain that has been used in developing a FMD vaccine. This strain has demonstrated good immune effects. The study selected the structural protein VP1 of strains AF/72 as the protein used to study epitopes because the structural protein VP1 contains the major antigenic sites of the foot-and-mouth disease virus and is the main antigen of the FMDV [41-43]. Sequence homology analysis of the AF/72 strain structural protein VP1 showed that, compared with the FMDV A/NM/XZ/64 (AJ131664) strains, A/NM/EL/60 (AJ131664) strain and GS/LX/62 (AJ131666) strain, which are isolated in China, showed there was a nucleotide homology of more than 97%. Additionally, the nucleotide sequences of the zones of the two identified CTL epitopes did not change. Therefore, the two CTL epitopes identified in the experiment are representative.

The successful use of biology software for prediction was considered when targeted epitopes were selected in the experimental design. Additionally, FMDV molecular biology characteristics were rarely determined, and the antigen epitope on the VP1G-H ring that had previously been identified were not identified in these experiments. This suggests that the characteristics of this virus's molecular biology and previous study results should be considered on the basis of software prediction in future epitope analysis tests.

## Conclusions

These experiments predicted 30 potential CTL epitopes on structural protein VP1 through bioinformatics, thus reducing the scope for the identification of potential epitopes and improving the accuracy of the experiments. Immunological experimental techniques were utilized to carry out the *in vitro *identification of the screened candidate epitopes that had been predicted. Finally, two murine H-2d restricted cytotoxic T cell epitopes on the FMDV VP1 structural protein were identified, including H-2Kd restricted T cell epitopes pK1 (AYHKGPFTRL) and H-2Dd restricted T-cell epitope pD7 (TGESADPVTT) from 106 to 115 amino acids and from 4 to 13 amino acids on VP1, respectively. This study provides direct evidence demonstrating the need for further clarification of the FMDV T cell epitopes. Additionally, it establishes research methods for studying other viral T cell epitopes.

## Materials and methods

### Virus and mouse

FMDV AF/72 strains were separated, identified and stored by our laboratory. Female SPF level six-week-old BALB/c mice were purchased from Lanzhou Institute of Biological Products and were fed in clean level environment.

### VP1 Antigen Protein Preparation

FMDV virus strain genomic group RNA was taken as a template. We used a pair of primers (upstream primer: 5'-CACAAATGTACAGGGATGGGT-3' and downstream primer: 5'-GACATGTCCTCCTGCATCT-3'). Complete nucleotide sequence of VP1 was obtained through reverse transcription and PCR extension. The recovered PCR products were connected to pGEM-T easy vector. The competent cell JM109 was transformed, and the plasmid was extracted after blue-white screening; moreover, the clone, which was identified as positive through PCR and whose enzyme was cut, was sent to Dalian TaKaRa Biotechnology Co., Ltd for sequencing.

We followed the E. coli codon bias to reform codons of wild-type VP1 genes and sent them to Shanghai Sangon Biotech Co., Ltd for synthesis under the conditions that there were no changes in the amino acid sequences of VP1 protein. This ensured that VP1 expression was accurate and efficient, thereby enabling the mouse immune program to be of a high level of quality. Synthesis genes were connected with vector pET28a and target protein expression was induced after the BL21 (DE3) host strain was transformed. Affinity chromatography columns were used for purification. SDS-PAGE and Western blotting was used for checking the molecular size and immunogenicity of the VP1 protein after expression conditions were varied compared with wild-type VP1 protein.

### Epitope Peptide Prediction and Preparation

The BIMAS program http://www-bimas.cit.nih.gov/molbio/hla_bind[44] was used for analyzing and predicting potential H-2Dd, H- 2Kd and H-2Ld restricted CTL epitopes on the VP1 protein according to the VP1 amino acid sequence. We identified ten H-2Dd, H-2Kd and H-2Ld restricted T cell epitopes that scored the highest. In total, 30 peptides were selected and sent to Shanghai Sangon Biotech Co., Ltd for synthesis. The purity of all synthetic peptides was higher than 95% based on quantifications from RP-HPLC measurements. The peptides were reconstituted with a certain amount of DMSO solution and RPMI1640 at a concentration of 1 mmol/L, and then the solution was packed and stored at -70°C.

### Mice Immunity

After affinity chromatography purification, VP1 protein was fully mixed with Freund's complete adjuvant with equal volume. SPF BALB/c mice were subcutaneously immunized with multi-point abdominal injections with an initial immunization dose of 100 μg/mouse. Booster immunizations were carried out 2 weeks later. Incomplete Freund's adjuvant was used during the booster immunization once every two weeks for an additional three times. VP1 protein was replaced with PBS for mice in the control group using the same immunization procedures. All animal studies were approved by the Review Board of Lanzhou Veterinary Research Institute, Chinese Academy of Agricultural Sciences (Permission number: SYXK-GAN-2004-0005). The mice used in this study were kindly bred during the experiment and euthanasia was carried out at the end of the experiment to reduce suffering.

### Preparation of Splenic Lymphocytes

After the immunizations were finished, mice were euthanized and spleens were harvested using sterile technique, and 3 mL of EZ-Sep ™ Mouse 1× lymphocyte isolation was placed in a 35 mm Petri dish with a 200 nylon mesh fixed on the dish. Following harvest, we made a single cell suspension of the mouse spleen by gently grinding the spleen using a syringe piston. The single cell suspension was maintained in 1640 medium at 0.5 mL. The cell suspension was centrifuged at 800 g for 30 minutes. The lymphocyte cell layer was isolated, washed in 10 mL of 1640 medium, and centrifuged at 250 g for 10 minutes. Supernatant was decanted and the cells were resuspended in serum-free media. Lymphocytes were resuspended in 1640 medium at a cell density of 10^7 ^cells/mL and were cultured at 37°C in 5% CO2.

### T Cell Proliferation Assay

One hundred microliters of the prepared lymphocyte cell suspension (10^7 ^cells/mL) was added to each well in a 96-well plate. Following the plating of the cell suspension, the synthetic peptide (30 μg/mL) was added with a final volume of 200 μL/well. Three parallel wells were made for each peptide section. RPMI 1640 medium was added in negative control wells without an additional stimulus. ConA was used for stimulation of the positive control group. Additionally, three parallel wells were set up with the final volume of 200 μL/well. The cultures were incubated at 37°C in a 5% CO2 incubator for 72 h. We added 20 μl CellTiter 96^® ^AQueous One Solution Reagent to each well 4 hours before the end of the culture. Following the addition of this reagent the cells were cultured at 37°C in a 5% CO2 incubator. After incubation, the absorbance was checked at 490 nm. The mean of the three duplicate wells (x ± s) was calculated, and the results were expressed as the stimulation index (SI) (SI = A_490 _mean of experimental group/A_490 _mean of negative control group). A positive result on the stimulation index was considered if SI ≥ 2.

### IFN-γ ELISPOT Experiment

PBS diluted coating antibody (50 μL) was added to each well on the PVDF 96-well plate. The plate was coated overnight at 4°C. The coating liquid was decanted and the plate was washed twice with PBS. The plate was dried on the sterile absorbent paper following the final wash. PBS (1×) diluted blocking solution was added (200 μL/well) and sealed at 37°C for 1 h. The blocking solution was decanted. The pre-prepared suspension of lymphocytes (100 μl at a concentration of 10^7 ^cells/100 μl) was added to each well. Synthetic peptides (10 μl, with a final concentration of 30 μg/mL) were added to the test group with three parallel wells prepared for each peptide. ConA (10 μg/mL) was added to the positive control group, but the negative control group received no added stimulus. The plate was covered after all the samples were added. It was placed in a carbon dioxide incubator and cultured for 24 h at 37°C. The cells and medium in the wells were decanted after incubation. Ice-cold deionized water (200 μL/well) was added. The plate was incubated in an ice bath for 10 min at 4°C. PBST (200 μL) was added in each well. After being washed five times, we added diluted biotinylated detection antibody (100 μL) to each well, which were incubated for 1 h at 37°C. Labeled detection antibody was incubated and washed. Then we added diluted HRP-avidin (100 μL) to each well, which was incubated at 37°C for 1 hour. Chromogenic reagent (100 μL) was added to each well after the above steps and washing. The plate was statically placed for 30 min at room temperature in a sun-shading place. After spots grew to a suitable size, the plate was washed with deionized water 2 times, and then the development process was stopped. The ELISPOT plate was placed in the automatic reading meter. The appropriate parameters were adjusted, spots were counted, and various parameters of the spots were recorded for statistical analysis. The results of the test were analyzed through variance analysis t tests. The differences were considered significant if P <0.05, and the difference was prominent if P <0.01.

## Abbreviations

FMDV: foot-and-mouth disease virus; IFN-γ: interferon-γ; STA: South African type; CTL:Cytotoxic T lymphocytes; MHC: major histocompatibility complex;SLA: Swine lymphocyte antigen;IPTG: Isopropyl β-D-1-thiogalactopy ranoside;SDS-PAGE: Sodium Dodecyl Sulfate - Polyacrylamide gel electrophoresis; ConA: Concanavalin A; SFC: Spot-forming cells;ELISPOT: Enzyme-linked immunospot

## Competing interests

The authors declare that they have no competing interests

## Authors' contributions

XSL and YLW conceived of the study. XSL Cloning and expressed VP1 genes, performed lymphocyte in vitro proliferation experiment and INF-γ ELISPOT experiments, analyzed the results and drafted the manuscript; YLW and YGZ supervised the research, analyzed the results and helped draft the manuscript; YZF and LP visualized the data. JLL, PZ, ZWZ and STJ assisted with data analysis and prepare the experiment. Manuscript is approved by all authors for publication.
